# Perioperative outcomes and economic impact of benign prostatic hyperplasia surgeries in Brazil’s public health system

**DOI:** 10.1186/s12913-025-13261-z

**Published:** 2025-09-30

**Authors:** Lucas Seiti Takemura, Ivan Kirche-Duarte, Gabriel Franco de Camargo Galindo, Felipe Arakaki Gushiken, Julio Silva Nogueira Luz, Jonathan Doyun Cha, Marcelo Langer Wroclawski, Luiz Vinicius de Alcantara Souza, Laercio da Silva Paiva, Gustavo Caserta Lemos, Bianca Bianco, Arie Carneiro

**Affiliations:** 1 Department of Urology, Hospital Israelita Albert Einstein, Av. Albert Einstein, 627, Bloco A1, sala 303 – Jardim Leonor, Sao Paulo/SP, Brazil; 2 Faculdade de Ciências Médicas da Santa Casa de São Paulo, São Paulo/SP, Brazil; 3 Faculdade de Medicina de ABC/Centro Universitário FMABC, Santo Andre/SP, Brazil

**Keywords:** Benign prostatic hyperplasia, Transurethral resection of the prostate, Simple prostatectomy, Public health system

## Abstract

**Background:**

Benign prostatic hyperplasia (BPH) is a common cause of lower urinary tract symptoms (LUTS) in aging men, and significantly affects their quality of life and productivity. In Brazil, where most of the population relies on the Public Health System (SUS), transurethral resection of the prostate (TURP) and simple prostatectomy (SP) are the primary surgical modalities. These procedures vary in cost-effectiveness, influencing clinical decisions and healthcare resource allocation. Therefore, we aimed to describe the perioperative outcomes of surgical modalities (TURP and SP) and the financial impact of these treatments in major Brazilian regions in recent years.

**Method:**

This ecological study utilized data from the Brazilian Public Health System database (DATASUS) from 2009 to 2022. The records of patients diagnosed with BPH and undergoing TURP or SP were analyzed across Brazil’s major geographic regions. The key outcomes included annual surgery volumes, patient demographic characteristics, hospitalization characteristics (e.g., length of stay and intensive care unit utilization), intrahospital mortality rates, and government reimbursements to hospitals. Statistical analyses included descriptive statistics, comparisons between the two techniques, and regression models to assess the temporal trends in mortality rates.

**Results:**

Over the 14-year period analyzed, Brazil recorded 204,358 BPH surgeries, with the Southeast region accounting for 46.56% of the procedures. TURP was the predominant procedure nationwide (61.44%), particularly in the higher-income regions. Perioperative outcomes favored TURP, with lower intrahospital mortality rates (0.25% vs. 0.55% for SP) and shorter hospital stays (median, 3 days vs. 5 days for SP). Both procedures resulted in decreasing mortality trends, although the differences were not statistically significant. Government reimbursements for hospitals were lower for TURP than for SP and did not keep pace with inflation during this period.

**Conclusion:**

This study underscores the prominent role of the Southeast region in BPH surgeries within Brazil's public health system and highlights TURP’s favorable perioperative outcomes of TURP over SP. It also showed a financial deficit in surgery reimbursements, which may impact the sustainability of the public health system.

**Supplementary Information:**

The online version contains supplementary material available at 10.1186/s12913-025-13261-z.

## Introduction

Benign prostatic hyperplasia (BPH) is the primary cause of lower urinary tract symptoms (LUTS) in men. It is estimated that 70% of men aged 60–69 years, and approximately 80% of men aged 70 years, are diagnosed with BPH [1]. On average, a person loses 7.3 working hours per year when they have the disease, and almost 10% of them report some work loss related to a healthcare visit for BPH, resulting in an economic impact [2]. The treatment of BPH is based on symptom severity and may include lifestyle modifications, medication, and/or surgery [3].

According to the last national census (Instituto Brasileiro de Geografia e Estatística, IBGE), in 2022, the Brazilian population consisted of 203 million people, 10.9% of whom were aged ≥ 60 years [4]. Approximately 70% of these individuals rely on the Public Health System (Sistema Único de Saúde, SUS), which is a tax-funded government entity. In this system, hospitals are paid according to the surgeries performed, which have specific codes and fixed costs. Given the limited resources at public services, patients with BPH and surgical indication have access mostly to two main surgical options: transurethral resection of the prostate (TURP) and simple prostatectomy (SP) [5].

The decision of which surgical technique should be offered to the patient is based on prostate volume. Although there is no established optimal threshold for prostate size, complication rates tend to increase as prostate size increases [6]. TURP is widely accessible, known for its effectiveness, and has historically been the benchmark method for the surgical treatment of BPH. SP is also a viable surgical option for men with a prostate size > 80 cc. Despite being more invasive than TURP, SP stands out for its effectiveness, safety profile, and long-term efficacy in treating larger prostate glands [7]. The potential complications of both procedures include bleeding, the need for blood transfusions, an extended hospital stay, urinary retention, the necessity for delayed bladder catheterization, urinary tract infections, and infections at the surgical site [8, 9].

To date, there are several epidemiological studies on BPH and its surgical treatment [10–12]; however, none have recently evaluated surgeries performed within Brazil’s public healthcare system on a nationwide scale. Therefore, we aimed to describe the perioperative outcomes of these two surgical modalities (TURP and SP) and the financial impact of these treatments in major regions of Brazil in recent years.

## Methods

### Data source

This was an ecological study that analyzed open data from Brazilian Public Health System database (DATASUS, https://datasus.saude.gov.br). The Big Data Department of Hospital Israelita Albert Einstein (São Paulo, Brazil) compiled medical records of patients with benign prostatic hyperplasia (ICD-10: N40) who underwent transurethral resection of the prostate (procedure code: 04.09.03.004–0) or simple prostatectomy (procedure code: 04.09.03.002–3) all over the country between 2009 and 2022. All data were de-identified when first collected.

The steps of collecting and selecting fields on the platform and later adjusting the tables were performed as described previously by Silva et al. (2024) [13]. The raw data went through an ETL (extract, transform, and load) process, created and described in the Applied Health Data Science (PCDaS) platform at Fiocruz (http://tabnet.fiocruz.br/dhx.exe?observatorio/tb_aih.def.), combining and integrating data from multiple sources into a single repository. They were extracted from the platform and enriched with the integration of other databases, generating the final dataset, organized in several.csv files organized by state and year.

The following outcomes were obtained for both procedures: number of surgeries performed per year, patient’s age at surgery, length of hospital stay, days in the intensive care unit (ICU), in-hospital mortality rate, type of surgery (elective or urgent), and total cost of hospitalization (in Brazilian real). Brazil and its major geographic regions (North, Northeast, Midwest, Southeast and South) were analyzed over the years and the outcomes were compared. Information regarding the costs presented in this study was the government payment to the hospitals for them to carry out these specific surgeries and not the actual costs of each patient treated individually. The costs were in Brazilian real (BRL) and compared to the national inflation trend over the years.

### Statistical analysis

A descriptive analysis of the data was conducted, with qualitative variables presented as absolute and relative frequencies. For quantitative variables, measures of central tendency, such as mean and median, and measures of dispersion, such as standard deviation and interquartile range, were used.

The relationship between in-hospital mortality rate, type of procedure (TURP or SP), and nature of hospitalization (elective or urgent) in different regions of Brazil was compared using the Chi-square test. Additionally, Poisson regression with robust variance was performed to analyze the risk of mortality associated with the type of procedure (with SP as a reference) and nature of hospitalization (with elective hospitalization as a reference).

The in-hospital mortality rate was calculated by dividing the number of deaths by the total number of BPH surgeries and multiplied by 100. To evaluate the in-hospital mortality rate by type of procedure (TURP or SP) during the period analyzed, time series rates were calculated using the Prais-Winsten regression model, as proposed by Antunes et al. [14]. This model allows the correction of first-order autocorrelation in temporally organized values. Thus, the following parameters were estimated: angular coefficient (β), corresponding probability (p), and the predictive capacity of the model (r2). Temporal analysis was performed by considering the location and year patterns with a 95% confidence interval. The directions of the indicator trends were analyzed and classified as increasing, decreasing, or stationary. The beta value of the regression was used as an indicator of this direction: a positive value denoted an increasing trend, whereas a negative value indicated a decreasing trend. When the beta value was zero, it suggested a stationary trend, implying no significant difference between the trend and zero, as described by Antunes et al. [14].

Data analysis was conducted using the"Data Analysis and Statistical Software for Professionals"(Stata) version 16.0®.

The inflation of the period between 2009 and 2022 was calculated based on the Broadened National Index of Costs and Prices to the Customer (IPCA, Índice Nacional de Preços ao Consumidor Amplo), the main inflationary index to gauge the costs of living. It is provided by the IBGE and is based on the current market price of the minimum amount of predefined items, including food, self-care, hygiene, and households, among others, needed to maintain a four-person family during a month with a gross income ranging from one to 40. These items are the core reference to calculate the above mentioned index. This is used for economic policies, taxes, financial interest rates, adjustment of salaries, and other costs and interests such as mortgages, fuel, and overall prices and costs of goods and services.

## Results

**Fig. 1:**
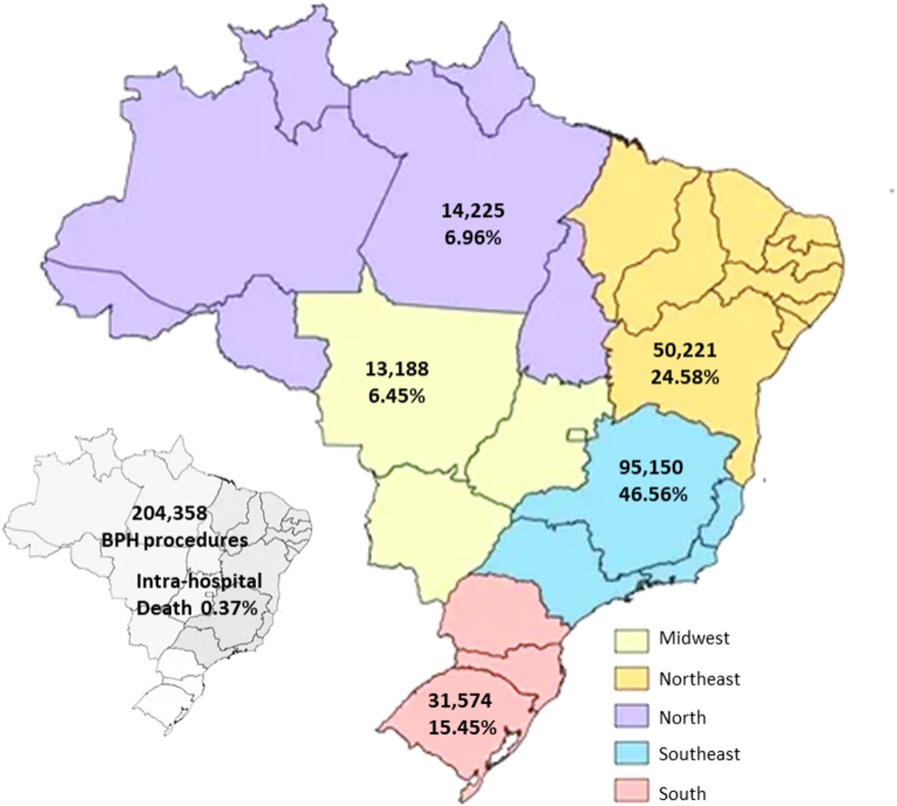
Number of surgeries for BPH in Brazil and its regions between 2009 and 2022

In Brazil, the national database registered 204,358 BPH procedures between 2009 and 2022 (Fig. 1). When analyzing the major Brazilian geographic regions, almost half of the surgeries (46.56%) occurred in the Southeast area, followed by the Northeast (24.58%), South (15.45%), North (6.96%), and Midwest (6.45%). Concerning the type of BPH procedure, there was a total of 125,567 TURPs (61.44%) and 78,791 SPs (38.56%). Table 1 shows that the Southeast, South, and Midwest regions performed more TURPs than SPs, whereas SP was predominant in the North and Northeast areas. Particularly in the Southeast region, the number of TURPs was 2.5 times the number of SPs. The number of procedures performed throughout the years was stationary (Supplementary Table 1).

**Table 1:** Distribution of surgeries for BPH and by type of procedure (simple prostatectomy and transurethral resection of the prostate) performed in Brazil between 2009 and 2022

Variables	Overall	Simple prostatectomy	Transurethral resection of the prostate
**Age range (n, %)**			
< 50 years old	3171 (1.55)	649 (0.82)	2522 (2.01)
50—59 years old	27573 (13.49)	8344 (10.59)	19229 (15.31)
60—69 years old	83699 (40.96)	32099 (40.74)	51600 (41.09)
70—79 years old	71182 (34.83)	29973 (38.04)	41209 (32.82)
≥ 80 years old	18733 (9.17)	7726 (9.81)	11,007 (8.77)
Age (mean, ± SD)	–-	69.07 (8.24)	67.63 (8.85)
**Procedures (n, %)**	204358 (100.0)	78791 (38.56)	125567 (61.44)
**Brazilian regions (n, %)**			
Southeast	95150 (46.56)	26753 (33.95)	68397 (54.47)
South	31574 (15.45)	12094 (15.35)	19480 (15.51)
Midwest	13188 (6.45)	5680 (7.21)	7508 (5.98)
North	14225 (6.96)	8773 (11.13)	5452 (4.34)
Northeast	50221 (24.58)	25491 (32.35)	24730 (19.69)
**Hospitalization (n, %)**			
Elective	165221 (80.85)	62558 (79.40)	102663 (81.76)
Urgency	39137 (19.15)	16233 (20.60)	22904 (18.24)
**In-hospital death (n, %)**	748 (0.37)	431 (0.55)	317 (0.25)
**Days of ICU**^**a**^	0 (0—0)	0 (0—0)	0 (0—0)
**Days of Hospitalization**^**a**^	3 (2—5)	5 (3; 7)	3 (2—4)
**Costs (BRL)**^**a**^	815.34 (620.08–1057.71)	1049.71 (1025.71—1149.61)	634.68 (618.68—711.48)

*ICU* Intensive care unit

^a^The values were showed as median and 25th and 75th Percentile. *SD * Standard Deviation

The mean age of the patients undergoing TURP was 67.6 years and that of those who underwent SP was 69.0 years. According to age stratification, 75.7% of BPH surgeries were performed in patients aged 60–79 years. Surgical treatment in patients aged < 50 years was rare (1.5%), and the majority underwent TURP. In contrast, 9.1% of surgeries were performed on octogenarians (Table 1).

**Fig. 2:**
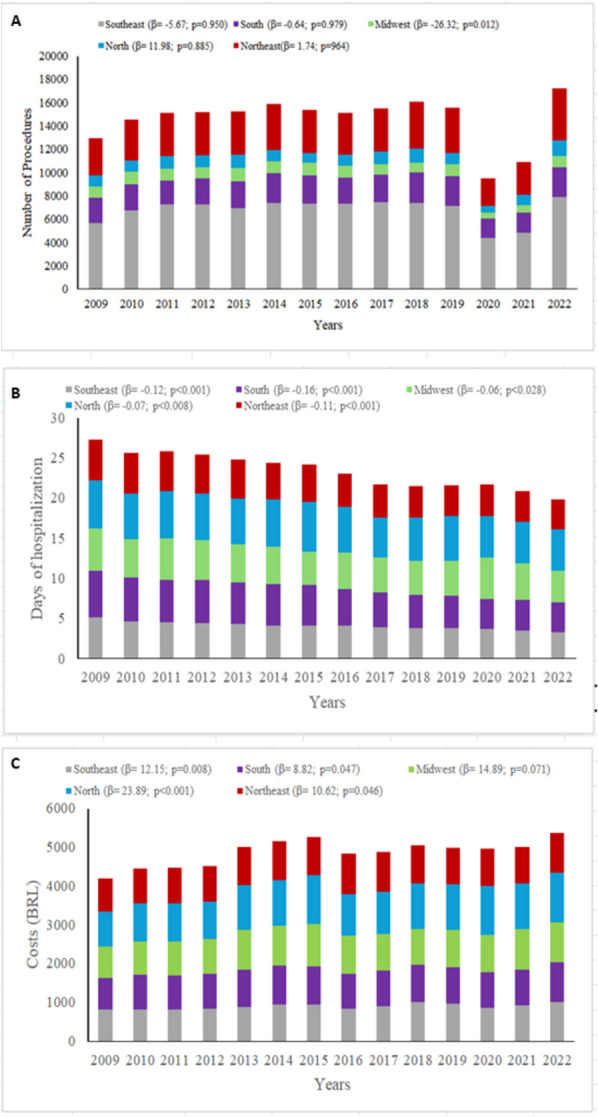
Variation in the number of procedures (**A**), days of hospitalization (**B**) and procedure costs (**C**) in each Brazilian region from 2009 to 2022

In 80.8% of the cases, surgeries were conducted during elective hospitalization. The median length of hospital stay for patients with BPH procedure was three days. For the same outcome, patients who underwent TURP had a median hospitalization time of three days, whereas those who underwent SP had a median of five days for the same outcome (Table 1). There was a decreasing trend in the length of hospital stay throughout the study period in all Brazilian regions (Fig. 2 and Supplementary Table 1). The median number of days spent in the ICU for both procedures was zero (Table 1).

**Fig. 3:**
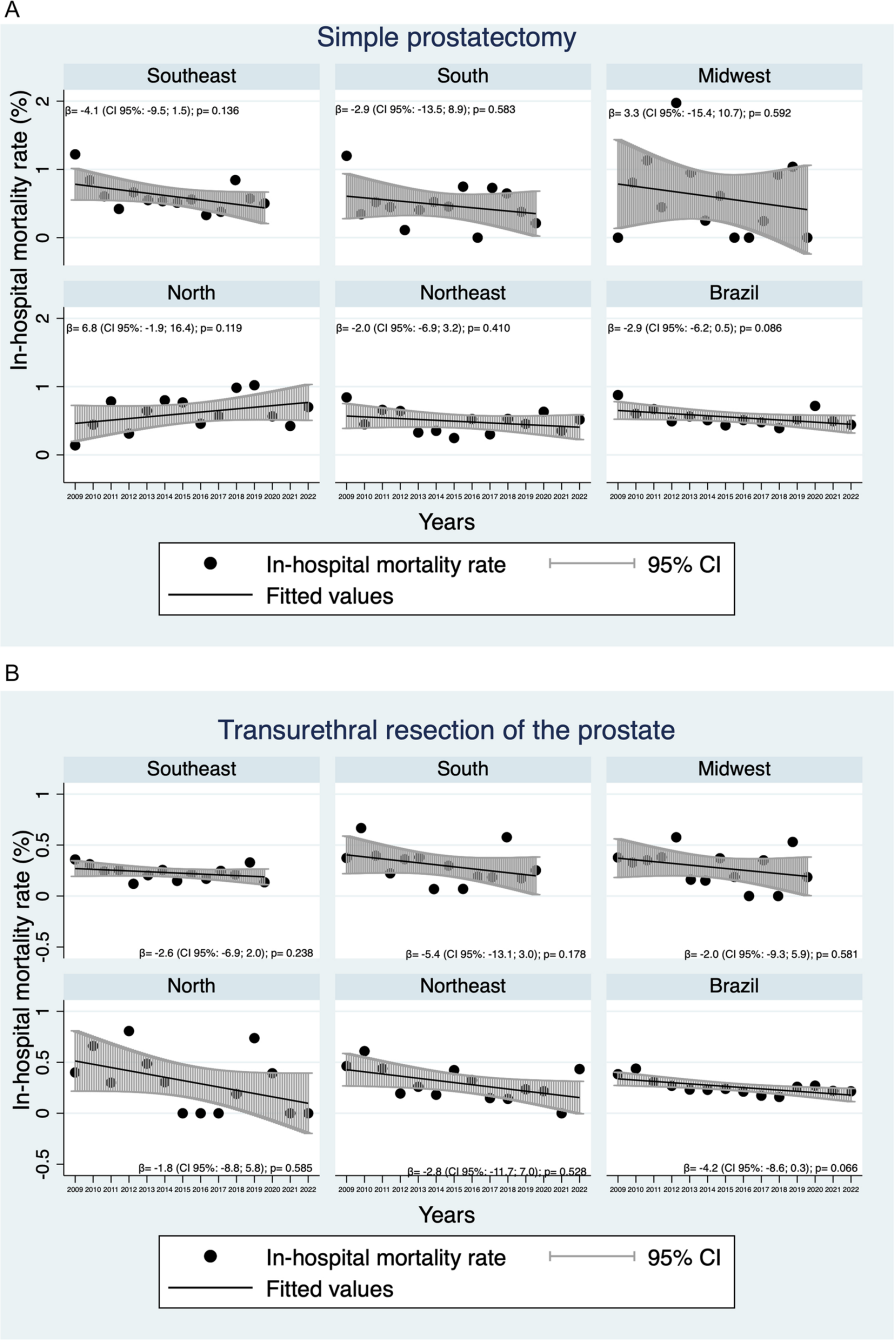
Temporal trend of intra-hospital mortality rate according to (**A**) Simple prostatectomy and (**B**) Transurethral resection of the prostate for the treatment of BPH in Brazil and its regions between 2009 to 2022

The in-hospital mortality rate of BPH surgery in Brazil is 0.37% (Fig. 1). Analyses of the risk of death by type of procedure showed that TURP had a statistically significant lower in-hospital mortality rate when compared to SP (RR: 0.46; CI: 0.39–0.53) (Table 2). All Brazilian regions showed the same findings when analyzed separately. Both TURP and SP showed a decreasing tendency with respect to mortality outcomes, although this did not reach statistical significance (Fig. 3).

**Table 2:** Analysis of intra-hospital mortality rate by type of BPH surgery (TURP and SP) and by type of hospitalization performed in Brazil and its regions between 2009 and 2022

Brazilian regions	Type of Procedure	Intra hospital death (n, %)	Brut RR (95% CI)	p*	Type of hospitalization	Intra hospital death (n, %)	Brut RR (95% CI)	p*
**Southeast**	SP	162 (0.61)	0.37 (0.29; 0.45)	0.001	Elective	241 (0.31)	1.40 (1.07; 1.82)	0.012
	TURP	152 (0.22)			Urgency	73 (0.43)		
**South**	SP	57 (0.47)	0.62 (0.43; 0.89)	0.011	Elective	82 (0.34)	1.19 (0.79; 1.79)	0.399
	TURP	57 (0.29)			Urgency	32 (0.41)		
**Midwest**	SP	34 (0.60)	0.49 (0.28; 0.84)	0.009	Elective	44 (0.41)	1.20 (0.64; 2.28)	0.566
	TURP	22 (0.29)			Urgency	12 (0.49)		
**North**	SP	54 (0.62)	0.44 (0.25; 0.79)	0.006	Elective	57 (0.51)	0.77 (0.41; 1.44)	0.414
	TURP	15 (0.28)			Urgency	12 (0.39)		
**Northeast**	SP	124 (0.49)	0.59 (0.44; 0.79)	< 0.001	Elective	160 (0.39)	1.01 (0.70; 1.45)	0.968
	TURP	71 (0.29)			Urgency	35 (0.39)		
**Brazil**	SP	431 (0.55)	0.46 (0.39; 0.53)	< 0.001	Elective	584 (0.35)	0.46 (0.40; 0.53)	< 0.001
	TURP	317 (0.25)			Urgency	164 (0.42)		

*RR* Relative Risk, *95% CI* Confidence Interval, *SP* simple prostatectomy, *TURP* transurethral resection of the prostate

^*^Poisson Regression Test with Robust Variance

Finally, the amount of money received by hospitals to perform BPH surgeries was BRL 838.58 in 2009 and BRL 1,075.97 in 2022. The correction index in the studied period using IPCA was 2,24 and the corresponding percentage value was 123,79%, the inflation-adjusted value would be BRL 2,365.54, representing a devaluation of 35% in the costs received by the hospitals.

## Discussion

This was a time series analysis comparing the surgical outcomes of TURP and SP for BPH in Brazil and its major regions from 2009 to 2022. Our data showed that the Southeast region concentrates the majority of BPH surgical procedures. We also found that TURP was predominant over SP in higher-income regions in Brazil [15]. This is likely due to easier and earlier access to healthcare centers in these locations, leading to a prompt diagnosis of BPH. Patients diagnosed earlier are more likely to have a prostate volume of less than 80 cc, favoring the TURP approach [5].

Another factor that could account for the distribution of procedures in our study was the availability of devices and technology required for TURP compared with SP. Performing TURP involves acquiring a video set, resectoscope, energy source (monopolar or bipolar), and resection loop in addition to the basic surgical material. These devices and technologies depend on the hospital resources and infrastructure accessibility. Consequently, lower-income regions might find it more feasible to afford SP than TURP despite SP's higher mortality rates and longer hospital stays ​[5, 16].

When analyzing the days of hospitalization for BPH procedures over the 14-year period, the Southeast region had the lowest average rate of hospital stay (4.17 days), while the North region had the highest results regarding this outcome (5.59 days). Our findings are in accordance with previous studies showing that institutions with a high volume of BPH surgeries tend to have a shorter length of hospital stay ​​[5]. Regarding days in ICU, both procedures had extremely low rates, making it difficult to draw any meaningful comparisons. Barbosa et al. [5] also showed that the days of ICU stay were not statistically different between low- and high-volume centers for TURP or SP.

Nationally, the mortality rate stands at 0.25%, demonstrating its safety as a treatment for BPH. The Southeast region demonstrated the lowest mortality rate of 0.22% for TURP, whereas the other regions showed slightly higher rates. These findings corroborate those of Reich et al. [6], who conducted a multicenter prospective cohort study with 9,197 men and observed that postoperative deaths accounted for an overall TURP mortality rate of 0.10%. This study also found that the morbidity and mortality rates were closely related to resection weight, with the mortality rate increasing to 0.71% when the resection weight exceeded 60 g. For SP, the mortality rate in Brazil was 0.55%, with the lowest rate in the South region (0.47%). Our findings support those of Gilfrich et al. [17], who reported TURP mortality rates of 0.32% and 0.51% in SPs in a nationwide German health insurance database. Madersbacher et al. [18] conducted a study in Austria including 23,123 cases and reported higher 90-day mortality rates for TURP (0.7%) and SP (0.9%)​​.

The type of hospitalization significantly affected mortality rates. Elective TURP and SP resulted in lower mortality rates (0.35%) than urgent procedures (0.42%). This difference underscores the benefits of preoperative planning and patient optimization, which are more feasible for elective surgery. These results are consistent with those of a Bavarian study by Reich et al. [6], which demonstrated that planned procedures typically lead to better outcomes owing to preoperative optimization and controlled surgical environments.

The temporal analysis in this study indicated a general decline in TURP mortality rates over the years, pointing to improvements in surgical techniques, better perioperative care, and advancements in medical technologies. The decline in SP mortality rates is not as pronounced, suggesting that although laparoscopy and robotic surgery are emerging as minimally invasive alternatives, the availability of these modalities is still limited in Brazil, and the majority of cases are probably being treated using the open technique. Other studies have also reported decreased mortality rates after TURP and SP [19]. Eredics et al. [19] reported an approximately 20% reduction in the in-hospital mortality rates after TURP and SP within a decade.

It should be emphasized that these improvements in clinical outcomes occurred against the backdrop of economic challenges. From 2009 to 2022, inflation significantly affected healthcare costs in Brazil. Inflation increased, affecting the purchasing power of hospitals, making medical supplies and services more expensive [20]. Meanwhile, government reimbursements for BPH procedures have not kept pace with inflation, representing a devaluation of 35% of the costs received by hospitals. This financial deficit, caused by inadequate reimbursement rates, negatively affects hospital budgets and public health system sustainability [21].

While this study provides valuable insights into the public health scenario for BPH surgeries, its design comes with inherent limitations. First, time series studies are not capable of establishing causality.

Our database is also limited, as it lacks information on disease characteristics prior to surgery, does not specify the surgical technique used (e.g., monopolar or bipolar TURP, or whether simple prostatectomy was performed via open, laparoscopic, or robotic approach), and provides no specific details regarding the causes of in-hospital mortality. We presume that these deaths are in some way related to the surgical treatment, given that they occurred during the same hospitalization. As mentioned previously, the costs presented in this study refer to government reimbursements to hospitals for performing these procedures, and not to the actual costs incurred per individual patient, which may ultimately exclude expenses related to treatment complications. Finally, our findings may not be generalizable to the entire Brazilian population, given that approximately 30% are covered by private health insurance or receive medical care through the private sector.

## Conclusions

In Brazil, the Southeast region is responsible for the majority of BPH surgeries. Overall, TURP showed better perioperative outcomes than SP in terms of the intrahospital mortality rate and days of hospitalization. There was a downward trend in the mortality outcomes for both procedures, although statistical significance was not reached. Moreover, government reimbursements for BPH procedures have not kept pace with inflation over the last few years, which has negatively impacted the sustainability of the public health system. These findings contribute to decision-making in BPH management, with the aim of enhancing patient outcomes and optimizing nationwide healthcare resource allocation.

## Supplementary Information

Supplementary Material 1: Table 1. Number of BPH surgical procedures, days of hospitalization, and procedure costs in each Brazilian region from 2009 to 2022

## Data Availability

The data that support the findings of this study are available from the corresponding author upon reasonable request.

## Abbreviations

BPH: Benign prostatic hyperplasia (BPH)
BRL: Brazilian real (R$)
DATASUS: Brazilian Public Health System database (https://datasus.saude.gov.br)
IBGE: Brazilian Institute of Geography and Statistics (*Instituto Brasileiro de Geografia e Estatística*)
ICU: Intensive care unit
IPCA: Broadened National Index of Costs and Prices to the Customer (*Índice Nacional de Preços ao Consumidor Amplo*)
LUTS: Lower urinary tract symptoms
SP: Simple prostatectomy
SUS: Brazilian Public Health System (*Sistema Único de Saúde*)
TURP: Transurethral resection of the prostate
