# Supplementary material for: Perioperative outcomes and economic impact of benign prostatic hyperplasia surgeries in Brazil’s public health system
Source: BMC Health Serv Res. 2025 Sep 30;25:1225. doi: 10.1186/s12913-025-13261-z (PMC12482657; doi:10.1186/s12913-025-13261-z)
Supplement: Supplementary file 1 — Supplementary Material 1: Table 1. Number of BPH surgical procedures, days of hospitalization, and procedure costs in each Brazilian region from 2009 to 2022 [file 12913_2025_13261_MOESM1_ESM.docx]

**Supplementary Table 1.** Number of BPH surgical procedures, days of hospitalization and procedure costs in each Brazilian region from 2009 to 2022.

| **Years** | **Number of procedures** | | | | | **Days of hospitalization** | | | | | **Costs (BRL)** | | | | |
| --- | --- | --- | --- | --- | --- | --- | --- | --- | --- | --- | --- | --- | --- | --- | --- |
|  | **Southeast** | **South** | **Midwest** | **North** | **Northeast** | **Southeast** | **South** | **Midwest** | **North** | **Northeast** | **Southeast** | **South** | **Midwest** | **North** | **Northeast** |
|  | **Absolute frequency** | | | | | **Mean** | | | | | **Mean** | | | | |
| 2009 | 5,672 | 2,173 | 974 | 961 | 3,197 | 5.177 | 5.817 | 5.281 | 6.005 | 5.045 | 815.06 | 829.96 | 805.39 | 901.06 | 841.47 |
| 2010 | 6,749 | 2,222 | 1,105 | 982 | 3,483 | 4.671 | 5.412 | 4.766 | 5.682 | 5.0872 | 835.36 | 877.81 | 861.90 | 975.03 | 909.99 |
| 2011 | 7,290 | 2,032 | 1,012 | 1,099 | 3,720 | 4.592 | 5.237 | 5.166 | 5.891 | 4.989 | 826.04 | 869.074 | 874.25 | 990.65 | 915.83 |
| 2012 | 7,262 | 2,243 | 974 | 1,013 | 3,726 | 4.478 | 5.303 | 4.988 | 5.792 | 4.915 | 842.70 | 906.71 | 891.34 | 957.71 | 931.38 |
| 2013 | 6,962 | 2,282 | 1,150 | 1,185 | 3,670 | 4.291 | 5.225 | 4.778 | 5.702 | 4.786 | 878.66 | 965.33 | 1038.31 | 1136.76 | 993.09 |
| 2014 | 7,383 | 2,566 | 1,042 | 953 | 3,921 | 4.147 | 5.116 | 4.663 | 5.954 | 4.537 | 957.23 | 989.22 | 1044.49 | 1172.98 | 990.04 |
| 2015 | 7,351 | 2,416 | 1,058 | 881 | 3,679 | 4.099 | 5.105 | 4.134 | 6.206 | 4.606 | 956.42 | 972.97 | 1097.58 | 1250.88 | 991.70 |
| 2016 | 7,330 | 2,222 | 1,030 | 979 | 3,559 | 4.103 | 4.609 | 4.477 | 5.733 | 4.127 | 840.12 | 896.69 | 998.72 | 1064.44 | 1046.51 |
| 2017 | 7,438 | 2,370 | 914 | 1,104 | 3,668 | 3.955 | 4.267 | 4.4168 | 4.939 | 4.119 | 905.47 | 920.68 | 951.41 | 1073.49 | 1025.89 |
| 2018 | 7,422 | 2,587 | 827 | 1,240 | 4,026 | 3.842 | 4.156 | 4.240 | 5.386 | 3.885 | 1026.22 | 958.54 | 915.45 | 1177.19 | 969.93 |
| 2019 | 7,124 | 2,592 | 982 | 995 | 3,890 | 3.846 | 4.050 | 4.287 | 5.553 | 3.908 | 978.87 | 946.12 | 945.15 | 1179.01 | 948.40 |
| 2020 | 4,380 | 1,658 | 507 | 609 | 2,334 | 3.670 | 3.797 | 5.122 | 5.234 | 3.929 | 860.81 | 914.75 | 977.98 | 1255.96 | 958.80 |
| 2021 | 4,867 | 1,680 | 666 | 886 | 2,840 | 3.529 | 3.763 | 4.638 | 5.121 | 3.846 | 941.18 | 906.11 | 1042.06 | 1172.87 | 951.37 |
| 2022 | 7,920 | 2,531 | 947 | 1,338 | 4,508 | 3.291 | 3.741 | 3.974 | 5.168 | 3.719 | 1014.34 | 1024.26 | 1039.22 | 1275.60 | 1026.44 |
| **β (95% IC)** | -5.67 | -0.64 | -26.32 | 1.98 | 1.74 | -0.12 | -0.16 | -0.06 | -0.07 | -0.11 | 12.15 | 8.82 | 14.89 | 23.89 | 10.62 |
| **p-value** | 0.950 | 0.979 | 0.012 | 0.885 | 0.964 | <0.001 | <0.001 | 0.028 | 0.008 | <0.001 | 0.008 | 0.047 | 0.071 | 0.001 | 0.046 |
| **Tendency** | Stationary | Stationary | Stationary | Stationary | Stationary | Decreased | Decreased | Decreased | Decreased | Decreased | Increasing | Increasing | Stationary | Increasing | Increasing |
